# Can We Find Better Bronchodilators to Relieve Asthma Symptoms?

**DOI:** 10.1155/2012/321949

**Published:** 2012-10-02

**Authors:** Elizabeth A. Townsend, Peter D. Yim, George Gallos, Charles W. Emala

**Affiliations:** Department of Anesthesiology, College of Physicians and Surgeons, Columbia University, 650 W 168th Street, New York, NY 10032, USA

## Abstract

Bronchodilators are the first line therapy during acute asthmatic exacerbations to reverse airway obstruction primarily by relaxing airway smooth muscle. Only three categories of bronchodilators exist in clinical practice: *β*-adrenergic agonists, anticholinergics, and methylxanthines. Each of these categories have specific drugs dating back to the early 20th century, raising the question of whether or not we can find better bronchodilators. While caffeine, theophylline, atropine, and epinephrine were the first generations of therapeutics in each of these drug classes, there is no question that improvements have been made in the bronchodilators in each of these classes. In the following editorial, we will briefly describe new classes of potential bronchodilators including: novel PDE inhibitors, natural phytotherapeutics, bitter taste receptor ligands, and chloride channel modulators, which have the potential to be used alone or in combination with existing bronchodilators to reverse acute airway obstruction in the future.

## 1. Novel PDE Inhibitors

Caffeine and theophylline are two methylxanthines that have been used to treat asthma, although their exact mechanisms of action are still unknown [1, 2]. The bronchodilating property of theophylline is largely attributed to inhibition of phosphodiesterase (PDE) activity or the release of catecholamines, thereby increasing cAMP in airway smooth muscle [3]. In use since the 1930s, lack of specificity, a narrow therapeutic window, and negative side effects, plus the development of inhaled glucocorticoids, and short- and long-acting *β*_2_-agonists have decreased the clinical utility of theophylline [1, 4, 5]. However, the identification of airway-specific PDE4 subtypes and subsequent development of PDE4-selective inhibitors have resurrected this avenue as novel bronchodilators.

The newest PDE4 inhibitor approved for use in respiratory disease is roflumilast (Daxas) [6, 7]. In patients with COPD, roflumilast has shown significant improvement of FEV1 when taken in conjunction with long-acting *β*_2_-agonists or muscarinic antagonists [8]. In preclinical studies, administration of selective PDE4 inhibitors prevented bronchial hyperresponsiveness (BHR) in allergic mice, an *in vivo* model for asthma [9, 10]. Although no PDE4-selective inhibitor is currently approved for the treatment of asthma in the United States, similar studies showed that administration of PDE4 inhibitors attenuated BHR in patients with allergic asthma [11–13]. With improved specificity for PDE4 subtypes, PDE4-selective inhibitors (roflumilast, CDP840, MK-0359), have decreased side effects compared to theophylline although the therapeutic window is still narrow and still produces systemic effects due to oral administration. Development of inhaled PDE4-selective inhibitors has shown potential in reducing early- and late-phase asthmatic responses in mild allergic asthmatics [14]. Whether or not these improvements in FEV1 are due to bronchodilation of airway smooth muscle or anti-inflammatory effects are yet to be determined; however, inhalation will likely reduce plasma levels of drug and decrease side effects associated with oral delivery. Further work on developing PDE4 subtype-specific inhibitors (A–D) or combining various PDE isoform inhibitors (i.e., PDE1, 3, 7 with PDE4 inhibitors) [5] may increase the efficacy of targeting this signaling pathway in treating asthma, providing a new application for a longstanding bronchodilator.

## 2. Natural Phytotherapeutics

Of note, one PDE4-selective inhibitor, quercetin, is a naturally occurring flavonol found in fruits, vegetables, and tea leaves. Retrospective studies have shown increasing numbers of asthmatics self-treat their symptoms with herbal remedies [15, 16]. In many cases, the exact mechanisms of action of these natural botanicals are unknown; however recent work has focused on identifying the active constituents of herbal remedies and elucidating the signaling pathways involved in acute bronchodilation. Given the advances in PDE inhibition and the natural origin of many methylxanthines, many of these natural phytotherapies may possess PDE inhibitory action. 

Recently, natural plant products have received accolades for the treatment of cough, respiratory infection, and bronchospasm [17]. It is estimated that 10%–42% of asthmatics use herbal therapies to self-treat their asthma symptoms [16, 18]; however the efficacy and safety of most herbal therapies have not been scientifically evaluated [19]. The exact mechanism of action of most of these agents is unclear but may involve direct effects on airway smooth muscle, airway epithelium, airway nerves, inflammatory cytokines, and immune cells. Moreover, the formulations of these herbal compounds are made up of many individual bioactive compounds. As such, it is important to define both the positive and potential negative impacts of these individual compounds on the airway as well as explore the interaction of herbal therapies with existing asthma therapies (corticosteroids and *β*-agonists).

Extensive preclinical, animal, and human studies have demonstrated that antiasthma simplified herbal medicine intervention (ASHMI), an extract of 3 plants *Ganoderma lucidum* (Ling-Zhi), *Sophora flavescens* (Ku-Shen) and *Glycyrrhiza uralensis* (Gan-Cao), reduces lung inflammation, airway remodeling, and airway smooth muscle hyperresponsiveness [20–22]. A blinded randomized trial in 91 subjects with moderate to severe allergic asthma demonstrated that 4 weeks of oral ASHMI were nearly equivalent to oral prednisone in the improvement in FEV1, peak flows, serum IgE levels, and eosinophilia [23]. The safety and tolerance of oral ASHMI were confirmed in a dose escalation study [21]. These clinical studies were followed by a series of pre-clinical studies that sought to identify the mechanism(s) involved in the improvement of symptom and inflammatory profiles. Both chronic and acute beneficial effects of ASHMI were demonstrated on mouse lung inflammation and responsiveness. Six weeks of oral administration of ASHMI reduced inflammation and *in vivo* responses to acetylcholine [20, 22, 24]. Acute treatment of isolated tracheal rings with ASHMI from naïve or ovalbumin sensitized mice demonstrated reduced acetylcholine-induced contractions in *ex vivo* organ bath experiments [22]. A possible mechanism for these acute effects was elucidated in human airway smooth muscle cells that liberated prostaglandins in response to ASHMI [22], which could mediate relaxation through activation of G_s_-coupled EP2 or EP4 receptors [25]. Current research is focused on identifying the specific purified chemical constituents of ASHMI that mediate these chronic anti-inflammatory effects and acute airway smooth muscle relaxant effects.

Although PGE_2_ relaxes airway smooth muscle in many species and benefits of inhaled PGE_2_ have been shown in asthmatics, a specific agonist for the EP2 receptor failed to show benefit in human trials [26]. However, newer studies suggest that targeting the EP4 receptor in human airway smooth muscle may be an alternative therapeutic target in patients with asthma [27].

## 3. Bitter Tastants

Another potential therapeutic target in the treatment of bronchoconstrictive disease involves the bitter taste receptor family (TAS2R). Recently, both qRT-PCR analysis and immunofluorescence microscopy of human airway smooth muscle (ASM) cells revealed robust expression of several members of this G-protein-coupled receptor family (TASR-10, -14, and -31) and showed increases in intracellular calcium ([Ca^2+^]_*i*_) in response to subsequent exposure to bitter tastants, the agonists to these receptor subtypes [28]. Despite increasing ASM [Ca^2+^]_*i*_ via the same pathway (G*βγ*→ PLC*β*→ IP_3_R) shared by the classical contractile agonist acetylcholine, this group paradoxically found activation of TAS2R in ASM leads to a profound degree of ASM bronchodilation in both isolated ASM preparations as well as *in vivo* models of induced airway responsiveness. Interestingly, the magnitude of bronchodilation achieved by high-dose TAS2R agonists in many of these studies rivaled maximal *β*-agonist treatments and mechanistically was found to be cAMP- and PKC-independent. This group has recently extended this observation to show that in relevant models of *β*_2_-adrenoceptor desensitization, chloroquine-mediated TAS2R activation in ASM retains its pro-bronchodilatory effects, a finding of considerable clinical relevance given the well-described concern of *β*-agonist tachyphylaxis that occurs with repetitive *β*-agonism [29]. Yet, it should be noted that TAS2R activation in ASM can lead to desensitization via a GRK-mediated, *β*-arrestin pathway, which may limit its therapeutic usefulness as it is seen currently with *β*-adrenoceptor agonists [30].

Mechanistically, TAS2R activation in ASM is thought to achieve relaxation via a localized [Ca^2+^]-dependent activation of the large conductance Ca^2+^-activated K^+^ channel (BK_Ca_) leading to membrane hyperpolarization. While other investigators have challenged the notion that bitter tastant-mediated ASM relaxation is BK_Ca_-dependent [31], the evidence in human ASM suggests at least a partial role of the BK_Ca_ channel in what is likely a novel, multimodal mechanism leading to ASM relaxation [32]. The possibility of TAS2R activation in ASM (in the context of localized calcium release) leading to non-BK_Ca_-mediated ASM relaxation via yet undescribed pathways is another exciting prospect behind this work that may uncover other potent targets to facilitate relaxation not susceptible to GPCR tachyphylaxis.

## 4. Chloride Channel Modulators

Chloride channels are expressed on airway smooth muscle and have been shown to effect airway smooth muscle force [33] and cell length [34]. In 2005, Hirota et al., described attenuation of acetylcholine-induced contractions in ASM subsequent to calcium-activated chloride channel antagonism [33]. Additionally, activation of the ligand-gated chloride channel, GABA_A_, relaxed airway smooth muscle precontracted with the tachykinin, substance P [35]. In 2011, another ligand-gated chloride channel, the glycine receptor, was shown to relax airway smooth muscle contracted with a selective neurokinin 2 receptor agonist [36]. These and other studies have led to the understanding that chloride channels may play a significant role in the airway smooth muscle contraction and relaxation mechanisms.

Calcium-activated chloride channels have been described functionally; however, the true molecular identity of calcium-activated chloride channels have only recently been identified as belonging to the ANO or TMEM16 receptor family. The TMEM16 receptors are membrane proteins with 8 transmembrane domains shown to allow chloride flux in the presence of increasing calcium while possessing voltage sensitive activity. TMEM16A mRNA expression has been described in airway smooth muscle [37] and its function in other cell types has been described as contributing to membrane depolarization during calcium increases [38]. It has been hypothesized that acetylcholine- and caffeine-mediated release of calcium from the sarcoplasmic reticulum (SR) stimulates chloride efflux from the cell, leading to depolarization of the plasma membrane. Force studies in *ex vivo* airway smooth muscle preparations examining the effects of chloride channel antagonists, 5-nitro-2-(3-phenylpropylamino)benzoic acid (NPPB), and niflumic acid (NFA), showed a large attenuation of acetylcholine-induced contraction by NPPB while NFA failed to have an effect. In contrast, caffeine-induced contractions were inhibited by both NFA and NPPB [33]. The differential effects of these chloride channel antagonists may be due to the effects on calcium-activated chloride channels located on the SR versus the plasma membrane. Recently, members of the TMEM family were shown to be expressed on various intracellular compartments and not exclusively on the plasma membrane [39]. A possible mechanism of attenuated force generation in airway smooth muscle by calcium-activated chloride channel antagonism may be inhibition of chloride ion efflux during contractile agonist stimulus.

Ligand-gated chloride channels have been well described in the central nervous system with two families dominating the role as inhibitory inputs, GABA_A_, and glycine receptor channels. Both GABA_A_ and glycine receptors are expressed in ASM and possess functional roles in the modulation of airway smooth muscle tone generation [35, 36]. This inhibitory effect on ASM contraction may be attributed to a relative hyperpolarization of the membrane potential after it has surpassed the chloride reversal potential following exposure to a contractile stimulus. This opening of the chloride channels causes an influx of chloride ions leading to a relative membrane hyperpolarization, eliminating, or attenuating the electromechanical component of contraction. Additional studies have described the importance of specific GABA_A_ receptor subunits. GABA_A_ channels containing alpha4 or alpha5 subunits can be selectively targeted in airway smooth muscle resulting in effects on membrane potential and airway tone [40]. Pharmacotherapies that are not GABA_A_ subunit selective, such as the general anesthetic, propofol, have bronchodilatory capabilities [41]. Increased airway smooth muscle specificity will determine the viability of GABA_A_-related therapies as bronchorelaxants. GABA_A_ function in airway smooth muscle has been studied in both rodent and human *ex vivo* models, as well as *in vivo* rodent models [42, 43], producing strong evidence that this channel, once thought to be exclusively neuronally expressed, may have direct effects on airway tone.

In the last ten years, the existence of chloride channels in airway smooth muscle has been confirmed yet our current understanding of their mechanistic and functional roles remains incomplete. Although poorly mechanistically understood [44, 45] manipulation of chloride channels still remains a viable avenue of further research in the discovery of novel bronchodilators. Continued research will uncover the exact mechanisms that dictate the role for chloride channels in the balance of contraction and relaxation in the airway.

While bronchodilators will likely continue to be a mainstay of asthma therapy far into the future, the classical relaxant, *β*-agonist, is not without limitations. Receptor desensitization, *β*-agonist insensitivity, *β*-agonist refractory bronchoconstriction, and even death are all risks associated with prolonged use of traditional *β*-agonists. As such, it is important to continually investigate new therapeutics for the treatment of asthma; keeping in mind that acute bronchodilation is the first line therapy during an asthmatic episode. Here we have illustrated 4 novel potential therapeutics that show functional bronchodilatory properties in the airway owing to a variety of mechanisms. These novel compounds may augment existing *β*-agonist relaxant effects as in the case of PDE inhibitors or provide complementary avenues for relaxation when combined with current therapies.  Table 1 summarizes beneficial aspects of traditional *β*-agonists and these novel therapeutics as well as illustrating current limitations to implementing these novel bronchodilators. Interestingly, compounds that transiently elevate [Ca^2+^]_*i*_ such as phytotherapeutics, bitter taste ligands, GABA_A_ receptor ligands, and chloride channel antagonists subsequently lead to functional relaxation of airways. This is counterintuitive in the face of decades of research closely linking global cellular calcium and smooth muscle contraction thus necessitating a broader understanding of complex calcium dynamics within cellular microdomains. While the mechanism of action of these potential therapeutics is still under investigation, they open the door for assessing new therapeutics and mechanisms leading to bronchodilation.

## Figures and Tables

**Table 1:** Summary of benefits and limitations of novel bronchodilators.

Drug class	Benefits	Limitations
*β*-agonists	Rapid airway relaxation Selective for *β*_2_-AR; decreased systemic effects	Receptor desensitization Receptor downregulation Refractory bronchoconstriction Asthma-related death

PDE inhibitors	Increase cAMP generated endogenously Enhance *β*_2_-AR effects Selectivity for subtypes specific to lung	Oral delivery Complex dosing and metabolism Systemic side effects Potential adenylyl cyclase and/or *β*_2_-AR desensitization

Phytotherapeutics	Airway relaxation Acute and chronic effects Reduces inflammation and remodeling Increased patient compliance	Mechanisms of action are not clearly defined Potential interaction with other drugs Difficulty standardizing source and dosing

Bitter tastants	Novel target—may augment traditional therapies due to cAMP-independence	Mechanisms of action are not clearly defined

Chloride channel modulators	Novel target—may augment traditional therapies May address neuronal components of airway tone	Mechanisms of action are not clearly defined Method of delivery Interaction with airway epithelium (mucous production) Systemic side effects
